# Affective Temperaments and Suicidality in Patients with Bipolar Disorder

**DOI:** 10.1192/j.eurpsy.2022.140

**Published:** 2022-09-01

**Authors:** M. Luciano

**Affiliations:** University of Campania “Luigi Vanvitelli”, Department Of Psychiatry, Naples, Italy

**Keywords:** affective temperament, BIPOLAR, violent suicide, Suicide

## Abstract

Suicide is one of the leading causes of death in patients with Bipolar Disorder (BD). Several risk factors linked to suicide attempts in patients with BD have been identified, including a long duration of illness, untreated BD, female sex, positive history for suicide attempts, comorbidity with substance abuse or personality disorders, anxiety, depressive polarity and recent psychiatric inpatient care. Recently affective temperaments have been considered as possible factors for suicide in BD. While hyperthymic temperament is associated with a reduced risk of suicide attempts, cyclothymic, irritable, depressive and anxious temperaments are more represented in patients with a positive history of suicide attempts. Moreover, cyclothymic and irritable temperaments are highly connected with both aggression and impulsivity, which play a role in suicidal behaviours. Despite this evidence, the predictive role of affective temperaments on suicide behaviours is still poorly studied. In this contribution, we will report results of a study aiming at assessing the relationship between affective temperaments and personal history of violent suicide attempts, in 74 patients with BD. Violent suicide attempts were positively associated with cyclothymic temperament and inversely to hyperthymic one. BD-I patients and patients with a clinical history of rapid cycling were significantly more represented in the group of patients with a history of violent suicide attempts. Our results suggest the role of affective temperaments in the suicidality of patients with BD.

**Disclosure:**

No significant relationships.

